# 16S rRNA Gene Amplicon Sequencing of Contaminated Coastal Sediment Collected from the Taehwa River Estuary, South Korea

**DOI:** 10.1128/MRA.00230-21

**Published:** 2021-05-13

**Authors:** Hee-eun Woo, Junho Lee, Jong-Oh Kim, In-Cheol Lee, Seokjin Yoon, Kyunghoi Kim

**Affiliations:** a Department of Ocean Engineering, Pukyong National University, Busan, Republic of Korea; b Research Vessel *Nara*, Pukyong National University, Busan, Republic of Korea; c Institute of Marine Biotechnology, Pukyong National University, Busan, Republic of Korea; d Dokdo Fisheries Research Center, National Institute of Fisheries Science, Pohang, Republic of Korea; University of Southern California

## Abstract

The Taehwa River Estuary is one of the largest enclosed bays in east Korea. In order to understand the environment of the Taehwa River Estuary, the microbial diversity in the sediment of the estuary was investigated through 16S rRNA gene sequencing. The predominant phyla in all locations were *Proteobacteria* and *Bacteroidetes*.

## Abstract (precis)

The Taehwa River Estuary is one of the largest enclosed bays in east Korea. In order to understand the environment of the Taehwa River Estuary, the microbial diversity in the sediment of the estuary was investigated through 16S rRNA gene sequencing. The predominant phyla were *Proteobacteria* and *Bacteroidetes* in all locations.

## ANNOUNCEMENT

The Taehwa River (length, 48 km; basin area, 644 km^2^) is an urban river that flows through Ulsan, the largest industrial city in South Korea (1, 2). After the construction of industrial complexes and Ulsan Port, large amounts of industrial wastewater and domestic sewage began to flow into the Taehwa River, and the coastal environment deteriorated dramatically (3). Hence, a special law was established in 2000 by the Ministry of Oceans and Fisheries of South Korea for the management of water quality in the Taehwa River Estuary. It has been reported that the water quality has improved since the water quality management measures were instituted (4–6). Nevertheless, problems such as eutrophication and algal bloom have persisted in the Taehwa River Estuary (7). In this study, the microbial diversity characteristics of sediments were investigated for efficient pollution control in the Taehwa River Estuary.

In September 2019, samples were collected from the surface layer of sediment at five stations in the Taehwa River Estuary, using a Peterson grab sampler (Table 1). Following the manufacturer’s instructions, DNA was extracted from the collected sediments using a DNeasy PowerMax soil kit (Qiagen). A sequencing library was prepared using a Herculase II Fusion DNA polymerase Nextera XT index kit ver. 2 (Illumina) with the primers Bakt_341F and Bakt_805R targeting the 16S rRNA gene (V3 to V4 regions). High-throughput DNA sequencing (300 bp, paired ends) was performed using an Illumina MiSeq system at Macrogen, Inc. (Seoul, South Korea), and the numbers of raw reads are presented in Table 1. The raw reads were trimmed using CutAdapt ver. 1.11 (default settings) (8) to remove adaptor sequences and cleaned using FLASH ver. 1.2.11 (default settings) (9) to remove the reads with low-quality scores (Q < 20). QIIME ver. 1.8.0 (default settings) (10) was used to calculate the number of operational taxonomic units (OTUs).

**TABLE 1:** Summary data description of samples collected from the Taehwa River

Station	Coordinates	No. of raw reads	No. of OTUs	SRA accession no.
US.w1	34°44.812′N, 127°45.445′E	120,885	11,801	SRX10016429
US.w3	34°45.15′N, 127°45.852′E	142,973	21,525	SRX10016430
US.w4	34°45.009′N, 127°45.516′E	113,724	12,536	SRX10016431
US.w5	34°44.861′N, 127°45.249′E	106,043	13,042	SRX10016432
US.w7	34°44.695′N, 127°45.591′E	108,905	11,991	SRX10016433

**FIG 1:**
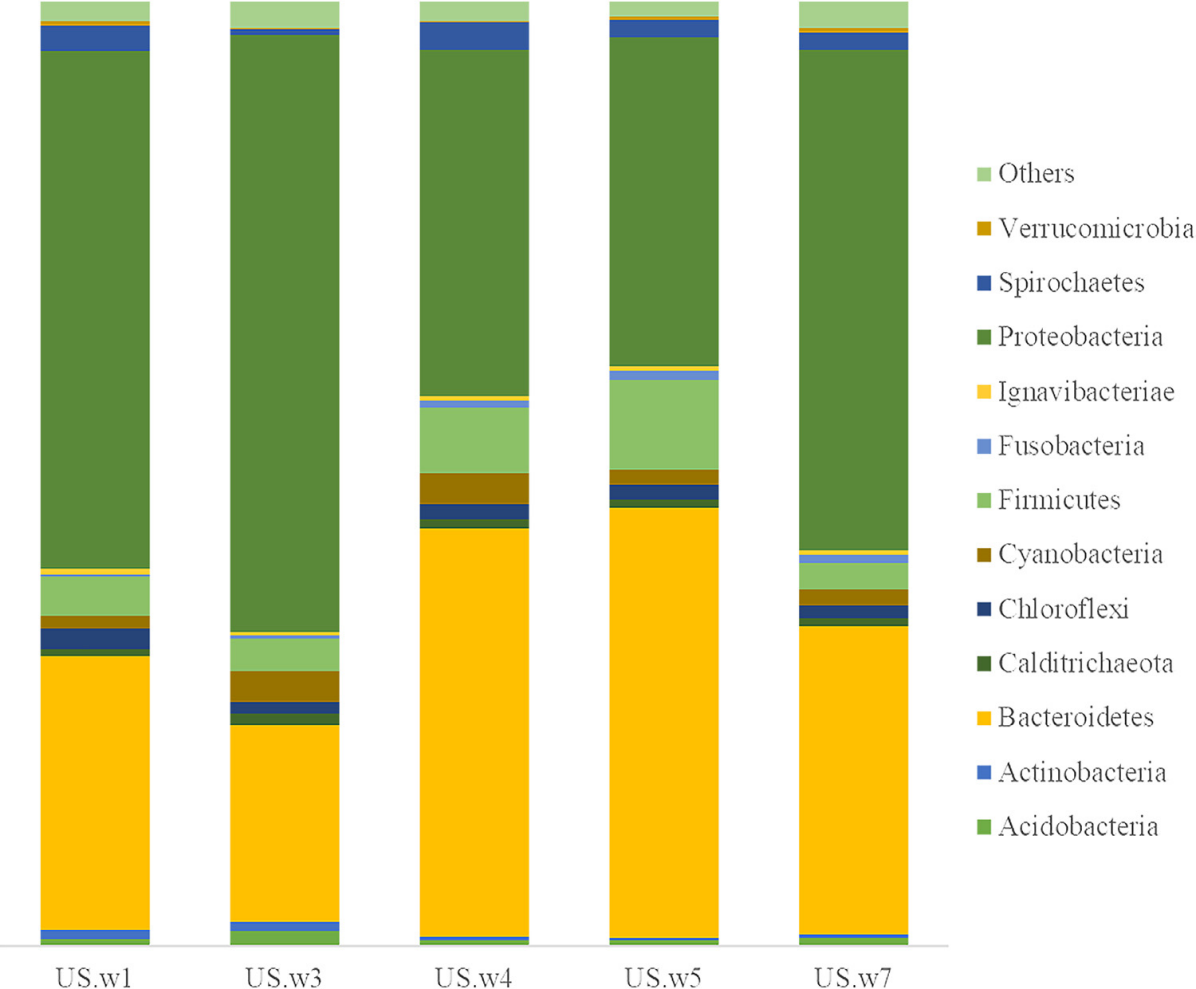
Relative abundance of bacterial phyla at each station.

The relative abundance of bacterial communities differed depending on the sampling location (Fig. 1). In all locations, the predominant phyla were *Proteobacteria* and *Bacteroidetes*, followed by *Firmicutes*, *Cyanobacteria*, and *Chloroflexi*. *Proteobacteria*, *Bacteroidetes*, and *Firmicutes* were the dominant phyla in the marine sediments (11). In particular, the predominance of *Firmicutes* may be due to the influence of anthropogenic activities (12).

### Data availability.

The amplicon sequences from this study are available in the NCBI database (BioProject accession number PRJNA699240).
